# Case Report: Behavioral Unresponsiveness in Acute COVID-19 Patients: The Utility of the Motor Behavior Tool-Revised and ^18^F-FDG PET/CT

**DOI:** 10.3389/fneur.2021.644848

**Published:** 2021-04-30

**Authors:** Sergiu Vijiala, Jean-Benoît Epiney, Jane Jöhr, Alessandro Pincherle, Marie M. Meyer, Renaud Du Pasquier, John O. Prior, Karin Diserens

**Affiliations:** ^1^Unit of Acute Neurorehabilitation Unit, Department of Clinical Neurosciences, Service of Neurology, Lausanne University Hospital (Centre Hospitalier Universitaire Vaudois), University of Lausanne, Lausanne, Switzerland; ^2^Department of Nuclear Medicine and Molecular Imaging, Lausanne University Hospital (Centre Hospitalier Universitaire Vaudois), University of Lausanne, Lausanne, Switzerland; ^3^Department of Clinical Neurosciences, Service of Neurology, Lausanne University Hospital (Centre Hospitalier Universitaire Vaudois), University of Lausanne, Lausanne, Switzerland

**Keywords:** COVID-19, SARS-CoV-2, neurological complications, unresponsiveness, PET, ^18^F-FDG

## Abstract

Along with the propagation of COVID-19, emerging evidence reveals significant neurological manifestations in severely infected COVID-19 patients. Among these patients admitted to the intensive care unit (ICU), behavioral unresponsiveness may occur frequently, yet, there are still only a few cases reported and with rare descriptions of their motor behavior after pathological awakening. Several hypotheses regarding central lesions in these patients are conceivable. Here, we describe two acute SARS-CoV-2- infected patients who developed neurological symptoms evoking the condition of clinical cognitive motor dissociation (CMD). This diagnosis could be confirmed first by clinical observation of a dissociation between preserved cognitive abilities and lack of initial motor interaction and second, by performing ^18^F- FDG PET imaging. Accurate diagnosis led to an appropriate neuro-rehabilitation regimen with long-term neuro-rehabilitation leading to an improved outcome for both patients.

## Introduction

COVID-19 is an infectious disease caused by severe acute respiratory syndrome coronavirus 2 (SARS-CoV-2), which is not only restricted to the pulmonary system. Cardiac, thromboembolic, hepatic, renal, gastrointestinal, ocular, endocrine, dermatological, and direct deleterious effects on the central and peripheral nervous systems have been described (1, 2). A retrospective study of 214 COVID-19 patients from China detailed various neurological manifestations in approximately one third of the cases, including acute cerebrovascular disease and impaired consciousness (3). The same study revealed that neurological manifestations in the ICU carried a poor prognosis. In a French case series of 58 consecutive severe acute-COVID-19 patients, encephalopathy with prominent agitation, confusion, and corticospinal tract signs were observed in almost two-thirds of the cases (4). Furthermore, in the 11 patients of this cohort who underwent perfusion imaging, all revealed bilateral frontotemporal hypoperfusion correlating with significant dysexecutive symptoms such as poorly organized motor responses to command in the follow-up.

In the acute stage, such critical damage to the motor output system confers the risk of underestimating the actual conscious awareness, as the patient may be overtly unable to interact even though his cognitive capacity is preserved. This condition is known as cognitive motor dissociation (CMD) (5) and is identified using functional brain imaging and electroencephalography. Such misdiagnosis may present serious consequences as real severe altered consciousness (i.e., true disorders of consciousness) carries unfavorable prognosis (6). Our group recently demonstrated that this particular condition might be also identified clinically (i.e., defined as clinical CMD, ^c^CMD) by means of the Motor Behavior Tool (MBT and its revised form, MBT-r) (7, 8). The use of this clinical tool as a complement to the Coma Recovery Scale-Revised (CRS-R) (9) allows the identification of subtle motor behavior undermined by the CRS-R, thereby uncovering patients with residual cognition and differentiating them from patients with real disorders of consciousness (DOC), the former having a better prognosis (10).

Complementary to the clinical evaluation, brain ^18^F-fluoro-deoxy-glucose positron emission tomography (FDG-PET) (11) is a noteworthy sensitive neuroimaging technique to detect brain function related to residual consciousness (12). Most FDG-PET studies have reported consistent, widespread reduced activity in patients with DOC, mainly in the pre-frontal, pre-motor, parietotemporal association areas, and the posterior cingulate cortex/precuneus, with evidence of impaired effective cortical connectivity between the pre-frontal, pre-motor, and posterior cingulate cortices and the thalamus (13). Very few studies have investigated cerebral metabolism in patients displaying covert cognition detected by paraclinical means, but some results indicated preserved metabolic patterns compatible with the presence of conscious awareness (14, 15). None however, investigated the metabolic activity in CMD identified solely by clinical evaluation.

Here, we describe two cases of acute patients infected with COVID-19 who developed neurological symptoms evoking the condition of clinical cognitive motor-dissociation (CMD). Both underwent brain ^18^F-FDG PET and required a long-term neuro-rehabilitation.

## Methods

The clinical and ancillary test descriptions were personally retrieved by the authors, who examined the patients. This report was conducted in compliance with the Swiss Federal Act on Research involving Human Beings, which waives ethical approval for case reports of less than five patients. Consent of the patient and/or his/her relatives for the re-use of personal data is therefore not required under Swiss Research legislation.

### Case Descriptions

#### Patient 1

**Figure 1:**
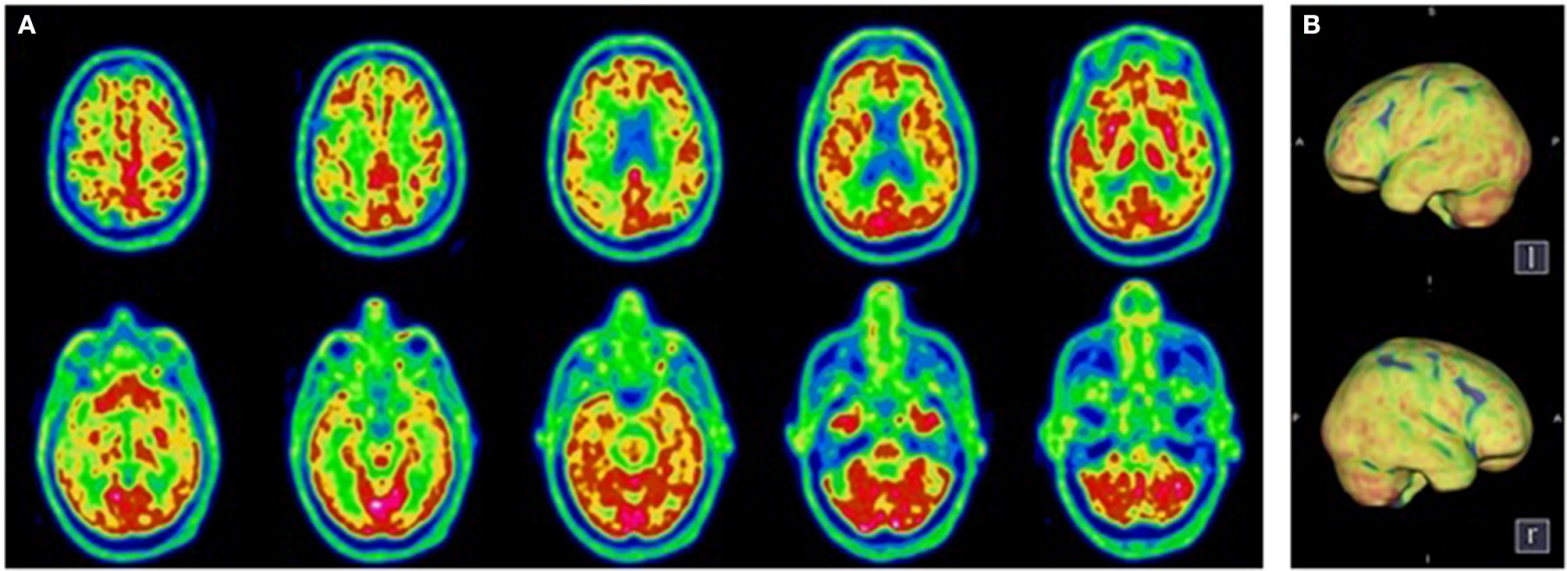
Transaxial **(A)** and Volume Rendered Brain **(B)** ^18^F-FDG PET. Diffuse cortical hypometabolism. Normal metabolism of the sub-cortical structures and the cerebellum.

A 78-year-old patient without psychiatric or oncologic history was admitted to the ICU with severe acute respiratory distress syndrome (ARDS) due to SARS-CoV-2 infection and needed endotracheal intubation for 25 days. He received hydroxychloroquine for 5 days and after extubation, the sedation was withdrawn. Two experienced physicians clinically assessed the patient at 7 days post-sedation withdrawal as he presented a pathological awakening with absent external responsiveness to stimulation and facial akinesia. According to the French version of the Coma Recovery Scale–Revised (16), he was classified as having unresponsive wakefulness syndrome (UWS) (Table 1). However, the MBT-r assessment categorized him as presenting with clinical cognitive motor dissociation (^c^CMD) with clear, subtle signs of conscious perception not considered by the CRS-R (i.e., attempt at visual pursuit, intentional defense gesture on painful simulation of the breast and an associated grimace). A routine electroencephalogram (EEG) ruled out a non-convulsive status epilepticus. Brain MRI was normal. The lumbar puncture was traumatic with 320 erythrocytes/mm^3^, without pleocytosis (<1 cell/mm^3^), normal lactate (2.28 mmol/l), a slightly diminished glucose ratio (0.39), the presence of oligoclonal IgG bands, which were identical in the serum and the CSF indicating rupture of the blood brain barrier consistent with systematic infection, negative SARS-CoV-2 and viral/bacterial pathogen PCR and normal ß-Amyloid (-42), hTau and Phospho-Tau (181P) levels. A brain ^18^F-FDG PET showed diffused hypometabolism of the cortical and subcortical regions of the two cerebral lobes, sparing partially the occipital cortex, the basal ganglia and the cerebellar cortex (Figure 1). Patient evolution was marked by a fluctuating hyperactive delirium treated by quetiapine, clonidine and melatonin. He was transferred to the internal medicine ward. His neurological symptomatology gradually improved. He regained voluntary control of his motor responses and followed simple commands, reaching the CRS-R criteria of recovery of consciousness. The overall swift rate of motor interaction recovery along with functional improvement, confirmed the preserved cognition as expected in CMD condition. The patient was transferred to a neuro-rehabilitation clinic, 44 days post-admission with a Glasgow Outcome Scale of three (indicating severe disability). He underwent neurorehabilitation for another 14 days attaining a Glasgow Outcome Scale of 4 (indicating moderate disability) and was able to return home to his wife.

**Table 1:** Patient demographics, clinical characteristics, and outcomes.

**ID**	**Sex**	**Age**	**Length of sedation (days)**	**Initial CRS-R diagnosis**	**CRS-R subscale scores**	**MBT-r classification**	**Time to hospital discharge (days)**	**GOS at discharge**
P1	M	78	25	UWS	A2V2M2O2C1Ar2	Clinical CMD	44	3
P2	M	61	65	MCS-	A2V2M3O2C1Ar2	Clinical CMD	105	3

*CRS-R, Coma Recovery Scale-Revised; UWS, unresponsive wakefulness syndrome; MCS–, minimally conscious state minus; MBT-r, revised Motor Behavior Tool; GOS, Glasgow Outcome Scale; The subscales for the CRS-R are Auditory Function (A), Visual Function (V), Motor Function (M), Oromotor Function (O), Communication (C), and Arousal (Ar); GOS score of 3 indicates Severe disability*.

#### Patient 2

**Figure 2:**
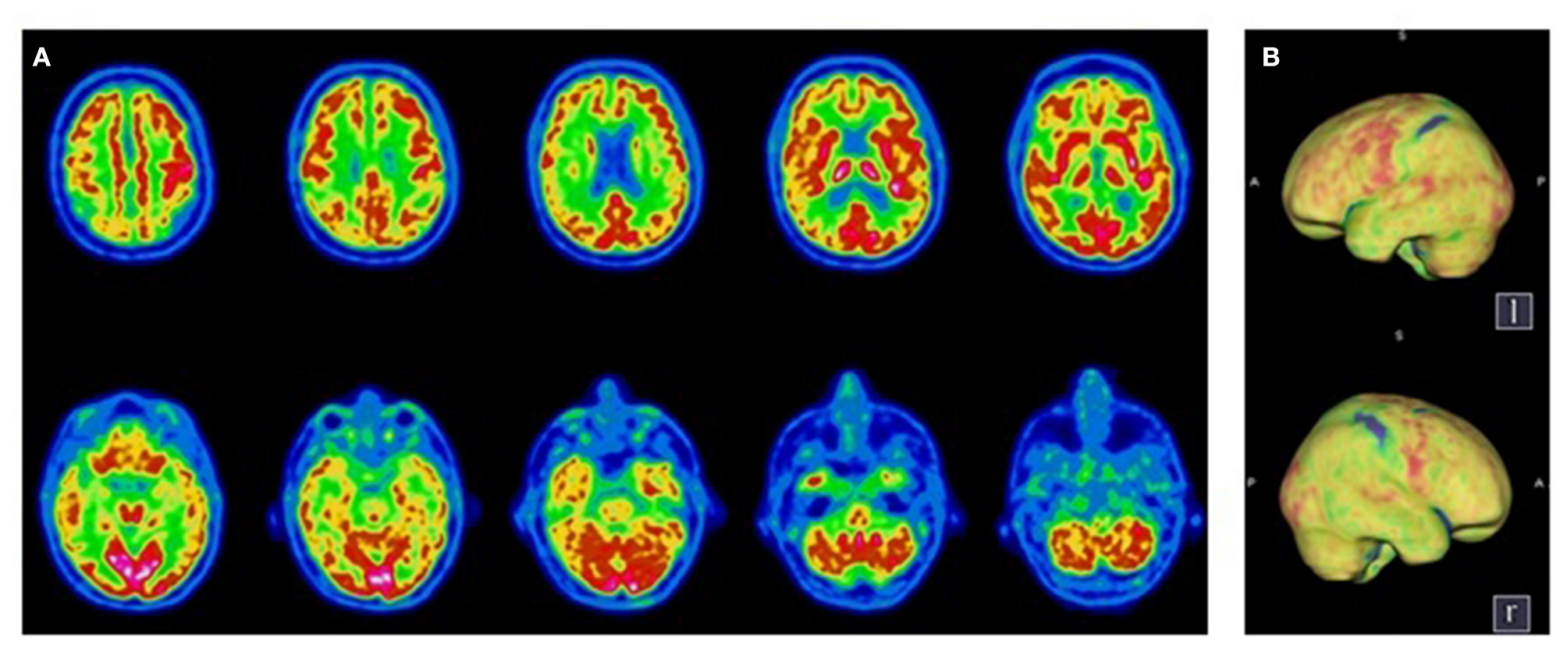
Transaxial **(A)** and Volume Rendered Brain **(B)** ^18^F-FDG PET. Diffuse cortical hypometabolism predominant in the right hemisphere, with a preservation of the primary sensorimotor areas. Normal metabolism of the sub-cortical structures and the cerebellum.

A 61-year old patient without known comorbidities was admitted to the ICU with severe ARDS due to SARS-CoV-2 infection and needed endotracheal intubation for 35 days with extracorporeal membrane oxygenation due to multiple complications. He received hydroxychloroquine and azithromycin for 5 days and one dose of tocilizumab. After 65 days, the sedation was withdrawn and the clinical evaluation 48 h later showed a pathological awakening with reduced behavioral evidence, severe dysfunction of the swallowing pattern with discoordination of the swallow motor circuit, and facial akinesia. According to the French version of the Coma Recovery Scale-Revised, the patient was classified as being in a minimally conscious state minus (MCS-) with no response to the command (Table 1), while the MBT-r assessment, considered the patient as presenting with clinical cognitive motor dissociation with the presence of tenuous motor signs (i.e., onset of visual pursuit on the vertical plane, and spontaneous intentional distal movements) not taken into account by the CRS-R, yet deemed as indicators of preserved cognitive abilities. A routine electroencephalogram (EEG) ruled out a non-convulsive status epilepticus, showing a moderate encephalopathy. Brain MRI was unremarkable. The electroneuromyography confirmed a critical illness polyneuropathy. A lumbar puncture could not be performed due to bilateral pulmonary embolism. A brain ^18^F- FDG PET showed a moderate hypometabolism in the frontal, temporal and parietal regions, sparing the motor, and pre-motor cortex (Figure 2). The neurological symptomatology improved gradually, the patient regained his ability to display overt motor behavior and responded systematically to commands, thus reaching the CRS-R criteria for recovery of consciousness. The overall functional/cognitive improvement confirmed the preserved cognitive abilities as expected in CMD condition and the patient was transferred to a neuro-rehabilitation clinic, 105 days post-admission with a Glasgow Outcome Scale of three (indicating severe disability). He underwent neurorehabilitation for another 69 days obtaining a Glasgow Outcome Scale of 4 (indicating moderate disability) and was able to return home.

## Discussion

The long-term outcomes of patients after severe COVID-19 are still unknown; nonetheless, a new emerging syndrome, Post Covid-19 Neurological Syndrome (PCNS) described by Wijeratne and Crewther shows myopathy and prolonged muscle weakness (17). Intensive care unit survivors have been shown to present a significantly lower health-related quality of life (18). The long stay in the ICU, often complicated by critical illness polyneuropathy/myopathy (19), cannot explain the global akinetic motor pattern observed in the two cases described here. Our patients presented severe swallowing disorders, facial akinesia, absence of oculomotricity and lack of motor interaction to stimulation, without any structural brain MRI signs of inflammatory, vascular, degenerative, or infectious encephalopathy.

The pathogenic mechanism explaining COVID-related neurological disorders and encephalopathy in particular, is the topic of intense discussion (2, 20–23). Indeed, it remains undetermined whether SARS-CoV-2 causes direct brain damages (possibly by affecting the olfactory nerves and migrating retrogradely) or whether the cause is indirect, due to an excessive inflammatory response (cytokine storm) or the trigger of an autoimmune response by the virus (24), but more evidence suggest a migration to the central nervous system via transfer across the blood-brain barrier (20, 23). Despite clear identifiable neurological associations of COVID-19 (25), the effect on motor interaction, which is an overt indicator of consciousness, is still undetermined. Some evidence suggests that coronaviruses may cause damage to the dopaminergic system. Earlier studies in patients with Parkinson's disease showed high anti-coronavirus antibody titers in the cerebrospinal fluid (26) and recently, SARS-CoV-2 was identified in frontal lobe tissue (27) using electron-microscopy. Furthermore, some COVID-19 patients have shown extended confusion after sedation withdrawal and impaired consciousness (28).

Consistent with these data, our hypothesis is that for the two patients, SARS-Cov-2 induced functional impairment in strategically localized areas of the executive motor network (i.e., frontal, pre-frontal). Indeed, the brain ^18^F-FDG PET in these patients showed a diffused hypometabolism, sparing the motor and pre-motor cortex but affecting the associative areas responsible for the integration of motor initiation and coordination, explaining the clinical picture.

Regarding prognostic implications, establishing whether a patient has preserved cognition/motor intent is of high significance; patients presenting with clinical CMD are likely to have a better prognosis and superior cognitive/functional outcomes (10), helping to select the most appropriate rehabilitation technique. The outcomes of the two cases described here, which was characterized by a rapid rate of cognitive and functional recovery but enduring executive and attentional disorders, confirmed the initial diagnosis of clinical CMD.

We recommend therefore, using the MBT-r as a simple and economic tool for distinguishing CMD patients from patients with real impairment of consciousness to avoid misdiagnosis in patients awakening from coma after severe COVID-19. This is crucial in the evaluation of COVID-19 patients, where exposure time of care-givers is correlated with the risk of infection (29). In addition to the clinical evaluation with the MBT-r, we recommend ruling out treatable causes by lumbar puncture and brain MRI. It is especially important to rule out stroke since patients with COVID-19 exhibit a higher risk of acute ischemic stroke compared with patients with other respiratory tract infections (30).

In cases of normal MRI results and behavioral unresponsiveness following severe COVID-19, brain ^18^F- FDG PET may also be used as a more robust technique for confirming the diagnostic hypothesis in selected patients (12). This exam is a complementary tool that can confirm the integrity of the structures responsible for voluntary movement, especially in patients with normal brain MRI or electromyography studies showing motor deficit only. A systematic exploration of all these particular COVID-19 cases using PET might be currently unrealistic due to the number of concerned patients and the theoretical risk of disease contaminating PET imaging departments (31); although we believe that imaging of COVID-19-positive patients can be practiced safely and patients who might benefit from this imaging should not be denied access, as demonstrated by our group (32).

A thorough but practical clinical examination investigating subtle positive signs, such as the MBT-r, complemented by ^18^F- FDG PET exploration in cases of other unremarkable brain imaging, would have a direct impact on patient care, potentially leading to better therapeutic interventions at an early stage. Indeed, establishing a rapid diagnostic procedure and reliable prognosis outcome is crucial for patients who might benefit from an early treatment (24). Above all, we recommend applying an early and intensive neuro-rehabilitation program for severe COVID-19 patients with behavioral unresponsiveness, which aims at maximizing patient function to achieve the highest possible level of independence (33, 34).

## Data Availability Statement

The raw data supporting the conclusions of this article will be made available by the authors, without undue reservation.

## Ethics Statement

Ethical review and approval was not required for the study on human participants in accordance with the local legislation and institutional requirements. Written informed consent for participation was not required for this study in accordance with the national legislation and the institutional requirements. Written informed consent was not obtained from the individual(s) for the publication of any potentially identifiable images or data included in this article.

## Author Contributions

SV, J-BE, JJ, AP, MM, RD, JP, and KD contributed to the conception and design of the review. SV, J-BE, JJ, and MM contributed to the literature review, drafting the text, and preparing the figures. All authors contributed to the article and approved the submitted version.

## Conflict of Interest

The authors declare that the research was conducted in the absence of any commercial or financial relationships that could be construed as a potential conflict of interest.

## References

[1] Gupta A Madhavan MV Sehgal K. Extrapulmonary manifestations of COVID-19. Nat Med. (2020) 26:1017–32. 10.1038/s41591-020-0968-3 32651579 PMC11972613

[2] Najjar S Najjar A Chong DJ Pramanik BK Kirsch C Kuzniecky RI. Central nervous system complications associated with SARS-CoV-2 infection: integrative concepts of pathophysiology and case reports. J Neuroinflammation. (2020) 17:231. 10.1186/s12974-020-01896-0 32758257 PMC7406702

[3] Mao L Jin H Wang M Hu Y Chen S He Q. Neurologic manifestations of hospitalized patients with coronavirus disease 2019 in Wuhan, China. JAMA Neurol. (2020) 77:683–90. 10.1001/jamaneurol.2020.1127 32275288 PMC7149362

[4] Helms J Kremer S Merdji H Clere-Jehl R Schenck M Kummerlen C. Neurologic features in severe SARS-CoV-2 infection. N Engl J Med. (2020) 382:2268–70. 10.1056/NEJMc2008597 32294339 PMC7179967

[5] Schiff ND. Cognitive motor dissociation following severe brain injuries. JAMA Neurol. (2015) 72:1413–5. 10.1001/jamaneurol.2015.2899 26502348

[6] Luauté J Maucort-Boulch D Tell L Quelard F Sarraf T Iwaz J. Long-term outcomes of chronic minimally conscious and vegetative states. Neurology. (2010) 75:246–52. 10.1212/WNL.0b013e3181e8e8df 20554940

[7] Pignat JM Mauron E Jöhr J De Keranflec CGH De Ville D Van Preti MG. Outcome prediction of consciousness disorders in the acute stage based on a complementary motor behavioural tool. PLoS ONE. (2016) 11:e0156882. 10.1371/journal.pone.0156882 27359335 PMC4928790

[8] Pincherle A Jöhr J Chatelle C Pignat JM Du Pasquier R Ryvlin P. Motor behavior unmasks residual cognition in disorders of consciousness. Ann Neurol. (2019) 85:443–7. 10.1002/ana.25417 30661258

[9] Giacino JT Kalmar K Whyte J. The JFK coma recovery scale-revised: measurement characteristics and diagnostic utility. Arch Phys Med Rehabil. (2004) 85:2020–9. 10.1037/t28455-000 15605342

[10] Jöhr J Halimi F Pasquier J Pincherle A Schiff N Diserens K. Recovery in cognitive motor dissociation after severe brain injury: a cohort study. PLoS ONE. (2020) 15:e0228474. 10.1371/journal.pone.0228474 32023323 PMC7001945

[11] Laureys S. The neural correlate of (un)awareness: lessons from the vegetative state. Trends Cogn Sci. (2005) 9:556–9. 10.1016/j.tics.2005.10.010 16271507

[12] Stender J Gosseries O Bruno MA Charland-Verville V Vanhaudenhuyse A Demertzi A. Diagnostic precision of PET imaging and functional MRI in disorders of consciousness: a clinical validation study. Lancet. (2014) 384:514–22. 10.1016/S0140-6736(14)60042-8 24746174

[13] Bodien YG Chatelle C Edlow BL. Functional networks in disorders of consciousness. Semin Neurol. (2017) 37:485–502. 10.1055/s-0037-1607310 29207410 PMC5884076

[14] Bodart O Gosseries O Wannez S Thibaut A Annen J Boly M. Measures of metabolism and complexity in the brain of patients with disorders of consciousness. NeuroImage Clin. (2017) 14:354–62. 10.1016/j.nicl.2017.02.002 28239544 PMC5318348

[15] Annen J Frasso G Crone JS Heine L Di Perri C Martial C. Regional brain volumetry and brain function in severely brain-injured patients. Ann Neurol. (2018) 83:842–53. 10.1002/ana.25214 29572926

[16] Schnakers C Majerus S Giacino J Vanhaudenhuyse A Bruno MA Boly M. A french validation study of the coma recovery scale-Revised (CRS-R). Brain Inj. (2008) 22:786–92. 10.1080/02699050802403557 18787989

[17] Wijeratne T Crewther S. Post-COVID 19 Neurological Syndrome (PCNS); a novel syndrome with challenges for the global neurology community. J Neurol Sci. (2020) 419:117179. 10.1016/j.jns.2020.117179 33070003 PMC7550857

[18] Myhren H Ekeberg Ø Stokland O. Health-related quality of life and return to work after critical illness in general intensive care unit patients: a 1-year follow-up study. Crit Care Med. (2010) 38:1554–61. 10.1097/CCM.0b013e3181e2c8b1 20473149

[19] Cummings MJ Baldwin MR Abrams D Jacobson SD Meyer BJ Balough EM. Epidemiology, clinical course, and outcomes of critically ill adults with COVID-19 in New York city: a prospective cohort study. Lancet. (2020) 395:1763–70. 10.1016/S0140-6736(20)31189-2 32442528 PMC7237188

[20] Gangadhar Zirpe K Dixit S Prabhakar Kulkarni A Sapra H Kakkar G Gupta R. Pathophysiological mechanisms and neurological manifestations in COVID-19. Indian J Crit Care Med. (2020) 24:975–80. 10.5005/jp-journals-10071-23592 33281325 PMC7689109

[21] Chang L Berger JR Kumar Bhoi S Feng J Yu H Sun T. Complications and pathophysiology of COVID-19 in the nervous system. Front Neurol. (2020) 11:573421. 10.3389/fneur.2020.573421 33343486 PMC7746805

[22] Kumar Jha N Ojha S Kumar Jha S Dureja H Kumar Singh S Shukla SD. Evidence of coronavirus (CoV) pathogenesis and emerging pathogen SARS-CoV-2 in the nervous system: a review on neurological impairments and manifestations. J Mol Neurosci. (2021) 17:20. 10.1007/s12031-020-01767-6 33464535 PMC7814864

[23] Wijeratne T Crewther S. COVID-19 and long-term neurological problems: challenges ahead with Post-COVID-19 Neurological Syndrome. Aust J Gen Pract. (2021) 50 (Suppl. 43). 10.31128/AJGPCOVID-43. [Epub ahead of print]. 33543150

[24] Dogan L Kaya D Sarikaya T Zengin R Dincer A Ozkan Akinci I. Plasmapheresis treatment in COVID-19-related autoimmune meningoencephalitis: Case series. Brain Behav Immun. (2020) 87:155–8. 10.1016/j.bbi.2020.05.022 32389697 PMC7204750

[25] Ellul MA Benjamin L Singh B Lant S Michael BD Easton A. Neurological associations of COVID-19. Lancet Neurol. (2020) 19:767–83. 10.1016/S1474-4422(20)30221-0 32622375 PMC7332267

[26] Fazzini E Fleming J Fahn S. Cerebrospinal fluid antibodies to coronavirus in patients with Parkinson's disease. Mov Disord. (1992) 7:153–8. 10.1002/mds.870070210 1316552 PMC7168426

[27] Paniz-Mondolfi A Bryce C Grimes Z Gordon RE Reidy J Lednicky J. Central nervous system involvement by severe acute respiratory syndrome coronavirus-2 (SARS-CoV-2). J Med Virol. (2020) 92:699–702. 10.1002/jmv.25915 32314810 PMC7264598

[28] Shaikh AG Mitoma H Manto M. Cerebellar scholars' challenging time in COVID-19 pandemia. Cerebellum. (2020) 19:343–4. 10.1007/s12311-020-01131-9 32301047 PMC7161715

[29] Canova V Lederer Schläpfer H Piso RJ Droll A Fenner L Hoffmann T. Transmission risk of SARS-CoV-2 to healthcare workers -observational results of a primary care hospital contact tracing. Swiss Med Wkly. (2020) 150:w20257. 10.4414/smw.2020.20257 32333603

[30] Karimi L Sales C Gillard Crewther S Wijeratne T. Acute ischemic stroke in SARS-CoV, MERS-CoV, SARS-CoV-2: neurorehabilitation implications of inflammation induced immunological responses affecting vascular systems. Front Neurol. (2020) 11:565665. 10.3389/fneur.2020.565665 33414753 PMC7783449

[31] Joob B Wiwanitkit V. 18F-FDG PET/CT and COVID-19. Eur J Nucl Med Mol Imaging. (2020) 47:1348. 10.1007/s00259-020-04762-6 32166511 PMC7080005

[32] Kamani CH Jreige M Pappon M Fischbacher A Borens O Monney P. Added value of 18F-FDG PET/CT in a SARS-CoV-2-infected complex case with persistent fever. Eur J Nucl Med Mol Imaging. (2020) 47:2036–7. 10.1007/s00259-020-04860-5 32418065 PMC7229432

[33] Pincherle A Jöhr J Pancini L Leocani L Dalla Vecchia L Ryvlin P. Intensive care admission and early neuro-rehabilitation. Lessons for COVID-19? Front Neurol. (2020) 11:880. 10.3389/fneur.2020.00880 32982916 PMC7477378

[34] Wade DT. Rehabilitation after COVID-19: an evidence-based approach. Clin Med. (2020) 20:359–65. 10.7861/clinmed.2020-0353 PMC7385804 32518105

